# Astragalus polysaccharide attenuates LPS‐related inflammatory osteolysis by suppressing osteoclastogenesis by reducing the MAPK signalling pathway

**DOI:** 10.1111/jcmm.16683

**Published:** 2021-06-02

**Authors:** Jianye Yang, Leilei Qin, Jiaxing Huang, Yuwan Li, Sha Xu, Hai Wang, Sizheng Zhu, Jiawei Wang, Bo Zhu, Feilong Li, Wei Huang, Xuan Gong, Ning Hu

**Affiliations:** ^1^ Department of Orthopaedics The First Affiliated Hospital of Chongqing Medical University Chongqing China; ^2^ Department of Orthopaedics Fuling Central Hospital of Chongqing Chongqing China; ^3^ Institute of Sports Medicine Beijing Key Laboratory of Sports Injuries Peking University Third Hospital Beijing China; ^4^ Department of Rehabilitation Medicine Banan Second People's Hospital of Chongqing Chongqing China; ^5^ Department of Orthopaedics Chongqing Dazu People's Hospital Chongqing China; ^6^ Outpatient Department Chongqing General Hospital Chongqing China

**Keywords:** Astragalus polysaccharide, inflammatory osteolysis, lipopolysaccharide, osteoclastogenesis

## Abstract

Bacterial products can stimulate inflammatory reaction and activate immune cells to enhance the production of inflammatory cytokines, and finally promote osteoclasts recruitment and activity, leading to bone destruction. Unfortunately, effective preventive and treatment measures for inflammatory osteolysis are limited and usually confuse the orthopedist. Astragalus polysaccharide (APS), the main extractive of Astragali Radix, has been widely used for treating inflammatory diseases. In the current study, in vitro and in vivo experimental results demonstrated that APS notably inhibited osteoclast formation and differentiation dose‐dependently. Moreover, we found that APS down‐regulated RANKL‐related osteoclastogenesis and levels of osteoclast marker genes, such as *NFATC1*, *TRAP*, *c‐FOS* and cathepsin K. Further underlying mechanism investigation revealed that APS attenuated activity of MAPK signalling pathways (eg ERK, JNK and p38) and ROS production induced by RANKL. Additionally, APS was also found to suppress LPS‐related inflammatory osteolysis by decreasing inflammatory factors' production in vivo. Overall, our findings demonstrate that APS effectively down‐regulates inflammatory osteolysis due to osteoclast differentiation and has the potential to become an effective treatment of the disorders associated with osteoclast.

## 1 INTRODUCTION

Skeletal metabolic homeostasis integrity is sustained and orchestrated by osteoclast interceded bone resorption and osteoblast interceded bone formation.^1^ An increase in osteoclast formation contributes to excessive bone remodelling and thus induces many osteolytic problems, including osteoporosis,^2^ aseptic loosening of prostheses,^3^ inflammatory osteolysis,^4^ rheumatoid arthritis^5^ and metastatic cancers.^6^ Among them, inflammatory osteolysis, characterized by excessive bone mass loss, is mainly attributed to bacterial products and/or implant‐derived wear particles and has become a common clinical complication. Bacterial products can stimulate inflammatory reaction and activate immune cells to enhance the production of inflammatory cytokines, and finally promote osteoclasts recruitment and activity, leading to bone destruction.^4^ Therefore, targeting the inhibition of the signalling pathway of osteoclasts' formation may be a tremendously vital strategy to treat osteolytic diseases. Unfortunately, effective preventive and treatment measures for inflammatory osteolysis are currently limited and usually cause orthopedist's confusions.

Lipopolysaccharide (LPS), a classic and potent endotoxin, is an important biologically active substance, which mainly localizes in the gram‐negative bacteria's outer cell membrane.^7^, ^8^ It has been evident by studies that LPS is a critical mediator of osteoclast differentiation in inflammatory osteolysis, accompanying chronic gram‐negative infection.^9^, ^10^, ^11^, ^12^ LPS is considered a potent inducer of inflammatory reactions and a pro‐inflammatory cytokine, stimulating macrophages, fibroblasts and other cells to secrete various pathogenic cytokines responsible for inflammation, such as IL‐1β, IL‐6 and TNF‐α. Moreover, LPS can also correspondingly activate the NF‐kB and MAPK signal pathways to facilitate osteoclastogenesis, resulting in destructive osteolysis.^9^, ^11^, ^12^ In addition to this indirect effect of LPS through inflammatory factors, it has been reported that LPS correspondingly activates TLR4 signalling cascades, further promoting the expression of CXCR4 and TRAF6, and ultimately likewise activates the MAPKs signalling pathway that subsequently leads to osteoclast overactivation.^10^, ^13^ Therefore, based on the rationale above, suppressing osteoclast formation and differentiation by inhibiting inflammation can be a promising strategy for treating LPS‐induced inflammatory osteolysis.

In recent years, plant‐derived natural products and their derivatives have been demonstrated to be valuable sources to explore new therapeutic approaches for clinical diseases due to their specific pharmacological activities. Astragali Radix (Huang Qi), the dried roots of *Astragalus membranaceus* (Fisch.) Bge. are one of the most well known herbal medicines widely used to treat distinct diseases in China.^14^, ^15^ Astragali Radix extracts contain numerous bioactive ingredients, among which Astragalus polysaccharide (APS) is one of the most critical bioactive extract components.^14^, ^16^ APS was found to exhibit various bioactivities, including antioxidant,^17^, ^18^ anti‐inflammatory,^14^, ^17^ immunoregulatory,^19^ hypoglycaemic^15^ and anti‐tumour activities.^20^ Na Dong et al demonstrated that APS alleviated the MAPK and NF‐kB signalling pathway gene expression levels and ultimately correspondingly reduced the production of inflammatory factors interleukin 6 (IL‐6), IL‐Iβ and TNF‐α induced by LPS.^21^ However, the effects of APS on inflammatory osteolysis caused by osteoclasts remain unrevealed.

In this work, it has been suggested that APS might potentially become a novel therapeutic approach for inflammatory osteolysis by suppressing osteoclast formation. We thus aimed to investigate the potential efficacy of APS on osteoclast‐associated osteolytic bone conditions and further elucidate the underlying molecular roles of APS in osteoclasts formation.

## 2 MATERIALS AND METHODS

### 2.1 Materials and reagents

The highly pure APS (>98%) was obtained from Solarbio Science & Technology Company, and a 5 mg/mL stock solution of APS was prepared in PBS. DMEM, α‐MEM and FBS were obtained from Thermo Fisher Scientific. Recombinant murine RANKL and M‐CSF were obtained from R&D Systems Reagents. The complete DMEM and α‐MEM induction mediums consisting of M‐CSF (25 ng/mL) and RANKL (50 ng/mL) were prepared for RAW264.7 cells and bone marrow‐derived monocytes (BMMs), respectively. Specific antibodies for c‐Fos, NFATc1, P38 phosphorylated (p) P38 and β‐actin were purchased from Cell Signaling Technology. ELISA kits for mouse IL‐6, IL‐1β, and TNF‐α and specific antibodies for CTSK, JNK, p‐JNK, ERK and p‐ERK were obtained from Bioss Antibodies. The TRAP Kit, Actin Cytoskeleton/Focal Adhesion Staining Kit and LPS were purchased from Sigma‐Aldrich. The Cell Counting Kit‐8 (CCK‐8) was purchased from Beyotime Biotechnology.

### 2.2 Cell culture

RAW264.7 cells were obtained from the American Type Culture Collection and cultured in a T25 flask with a complete DMEM consisting of FBS (10%), penicillin (100 U/mL) and streptomycin (100 µg/mL). Six‐week‐old C57BL/6J mice were used to isolate BMMs following the protocols consent by the Animal Ethics Committee of the Chongqing Medical University. The long bones were dissected to remove all soft tissues, and the bone marrow was flushed from the femur and tibia. Cells were incubated for 24 hours in α‐MEM supplemented with 25 ng/mL M‐CSF, 10% FBS, penicillin (100 units/mL) and streptomycin (100 mg/mL). Subsequently, non‐adherent cells were removed. After that, adherent BMMs were cultured in a T25 flask with a complete α‐MEM consisting of M‐CSF (25 ng/mL) in an incubator at a constant temperature of 37°C with 5% CO_2_.

### 2.3 Cytotoxicity assay

RAW264.7 and BMMs (5 × 10^3^ per well) were plated in 96‐well plates. After 24 hours pre‐incubation, the cells were administrated by APS (0, 1, 5, 10, 50 or 100 µg/mL) for 24 hours, 48 hours and 72 hours, respectively. Ten microliters of the CCK‐8 working solution were added afterwards. The plate was incubated at 37°C for another 2 hours avoiding light. A microplate reader (Multiscan Spectrum; Thermo Labsystems) was applied to determine the absorbance at 450 nm.

### 2.4 Osteoclastogenesis assays

RAW264.7 or BMMs (5 × 10^3^ per well) were placed into 96‐well plates for 12 hours and then cultured in the complete induction medium. The RAW264.7 or BMMs were administrated with APS or blank. The medium of cell culture was changed every two days. After a five‐day incubation, all the RAW264.7 or BMMs were fixed with 4% paraformaldehyde (PFA) for 30 minutes and washed with PBS in triplicate. A light microscope was then applied to count TRAP‐positive multinucleated cells (nuclei ≥3). TRAP staining was performed on the cells using a TRAP kit following the manufacturer's procedures. In the next moment, a light microscope was used again to count TRAP‐positive multinucleated cells (nuclei ≥3). As above, for LPS‐induced osteoclastogenesis, RAW264.7 and BMMs were cultured in the complete induction medium for 24 hours, then cultured in a complete medium consisting of LPS (100 ng/mL) and were treated with APS or blank PBS. In the end, the osteoclasts were counted.

### 2.5 In vitro bone resorption assay

To measure osteoclast activity, RAW264.7 (1 × 10^5^ per well) was cultured on 6‐well plates in the complete induction medium until the generation of mature osteoclasts. The identical numbers of mature osteoclasts (1 × 10^4^ cells per well) were placed into hydroxyapatite‐coated 96‐well plates (Corning Osteoassay). The induced cells were treated with APS or blank PBS. After a two‐day incubation, sodium hypochlorite was used to bleach the wells three times (10 minutes per cycle) to remove cells, followed by the resorbed area measurement. The percentage area of hydroxyapatite surface resorbed by the osteoclasts was quantified by utilizing ImageJ software (NIH).

### 2.6 Actin cytoskeleton and focal adhesion staining

RAW264.7 cells (5 × 10^3^ per well) were first cultured in 96‐well plates for 24 hours. The cells were then stimulated in the complete induction medium and treated with APS or blank PBS. After forming mature osteoclasts in the positive group without APS, the cells were then fixed with 4% PFA, permeabilized with 0.1% Triton X‐100 PBS, and blocked with 3% BSA in PBS. Based on the manufacturer's instructions, the cells were subsequently incubated with rhodamine‐conjugated phalloidin for 1 hour avoiding light for F‐actin staining and fluorescently conjugated secondary antibodies for 1 hour. In the end, the cells were rinsed with PBS. The nuclei were counterstained with DAPI and mounted for fluorescence microscopy. The multinucleated cells were quantified by implementing ImageJ software.

### 2.7 RNA isolation and RT‐qPCR

RAW264.7 and BMMs (1 × 10^5^ per well) were placed in 6‐well plates for 24 hours and then cultured in the complete induction medium. The cells were administrated with APS or blank PBS. After a five‐day (BMMs) or three‐day (RAW264.7) incubation, total RNAs were isolated from cells by utilizing TRIzol reagent (Thermo Fisher Scientific) based on the manufacturer's instructions. One microgram of total RNAs was applied to synthesize cDNA using reverse transcriptase (Takara Bio Inc). SYBR Premix Ex Taq II (Takara Bio Inc) was implemented to conduct qRT‐PCR in a PCR detection system (Bio‐Rad). The primer sequences are summarized in Table 1. The levels of the target genes were determined relative to the levels of GAPDH's messenger RNA (mRNA).

**TABLE 1:** The sequences of primers for qPCR

Gene	Forward	Reverse
*TRAP*	CACTCCCACCCTGAGATTTGT	CATCGTCTGCACGGTTCTG
*CTSK*	GAAGAAGACTCACCAGAAGCAG	TCCAGGTTATGGGCAGAGATT
*c‐FOS*	CGGGTTTCAACGCCGACTA	TTGGCACTAGAGACGGACAGA
*NFATc1*	CCCGTCACATTCTGGTCCAT	CAAGTAACCGTGTAGCTGCACAA
*DC‐STAMP*	CTAGCTGGCTGGACTTCATCC	TCATGCTGTCTAGGAGACCTC
*OC‐STAMP*	GGGCTACTGGCATTGCTCTTAGT	CCAGAACCTTATATGAGGCGTCA
*ATP6V0D2*	AGCAAAGAAGACAGGGAG	CAGCGTCAAACAAAGG
*OSCA*	GGTCCTCATCTGCTTG	TATCTGGTGGAGTCTGG

### 2.8 Western blot analysis

RAW264.7 and BMMs (1 × 10^5^ per well) were placed in 6‐well plates for 24 hours and then cultured with APS or blank PBS in the complete induction medium for five days (BMMs) or three days (RAW264.7). The RIPA lysis buffer containing a cocktail combination of protease inhibitor and phosphatase inhibitor was applied to extract total cellular proteins from the cells abovementioned. The obtained proteins were then resolved by SDS‐PAGE and electron transferred to PVDF membranes (GE Healthcare). All membranes were blocked in a TBST solution consisting of 5% non‐fat milk for 1 hour before incubating with primary antibodies overnight. The membranes were then rinsed with TBST in triplicate and immersed in the secondary antibody at room temperature for 2 hours. The reactivity of the antibody was then determined by an enhanced chemiluminescence reagent (GE Healthcare). The protein bands' images were captured by the Chemi Doc XRS+Imaging System (Bio‐Rad) followed by the analyses using the ImageJ software. β‐actin was applied as an internal control.

### 2.9 Intracellular reactive oxygen species detection

Intracellular ROS levels were investigated by a DCFDA cellular ROS detection assay kit (Abcam). BMMs (5 × 10^3^ per well) were cultured in 96‐well plates in the complete induction medium and were treated with APS for 72 hours. The cells were then incubated with DCFDA at 37°C, avoiding light, for 30 minutes. The images were taken by fluorescence microscopy.

### 2.10 Enzyme‐linked immunosorbent assay

For the mouse blood sample, the blood coagulated naturally at room temperature for 20 minutes and was centrifuged for about 20 minutes (2000 rpm) at 4°C. After that, the ELISA test was performed on the collected supernatant, and the corresponding ELISA kits were applied to determine the levels of IL‐6, IL‐1β and TNF‐α in mouse blood samples following the manufacturer's instructions.

### 2.11 LPS‐stimulated mouse model of calvarial osteolysis

Animal care and experimental protocols were established in line with the guidelines for Ethical Conduct in the Care and Use of Nonhuman Animals in Research by the American Psychological Association and were consent by the Animal Ethics Committee of the Chongqing Medical University. C57BL/6 mice (female, 6‐week‐old) were purchased from the animal centre in Chongqing Medical University. Mice were kept for at least five days prior to being recruited into the current studies and were randomized into four groups (four mice per group): (a) blank control group (PBS‐treated); (b) LPS control group (5 mg/kg); (c) APS low‐dose group (LPS 5 mg/kg and APS 50 mg/kg); (d) APS high‐dose APS group (LPS 5 mg/kg and APS 400 mg/kg). Mice were injected subcutaneously daily over the sagittal midline suture of the skull under mild anaesthesia for 14 days. The mouse blood samples were collected for ELISA analysis by excising the eyeballs under deep anaesthesia. All mice were euthanized, and their skulls were separated for micro‐CT scan. Subsequently, the skull samples were fixed with 4% PFA and decalcified with 10% EDTA for 2 weeks. Finally, H&E staining was performed, and the obtained results were used to determine inflammatory osteolysis in vivo.

### 2.12 Statistical analysis

Unless otherwise indicated, all experiments were carried out in triplicate independently. Data are exhibited as mean ± standard deviation. The Student's *t* test was applied to conduct difference analyses between the indicated groups. GraphPad Prism 8 (GraphPad Software) was used to carry out all the analyses. *P values less than .05, **P values less than .01 and ***P values less than .001 were determined to be statistically significant.

## 3 RESULT

### 3.1 APS did not display cytotoxic effects on RAW264.7 and BMMs

**FIGURE 1:**
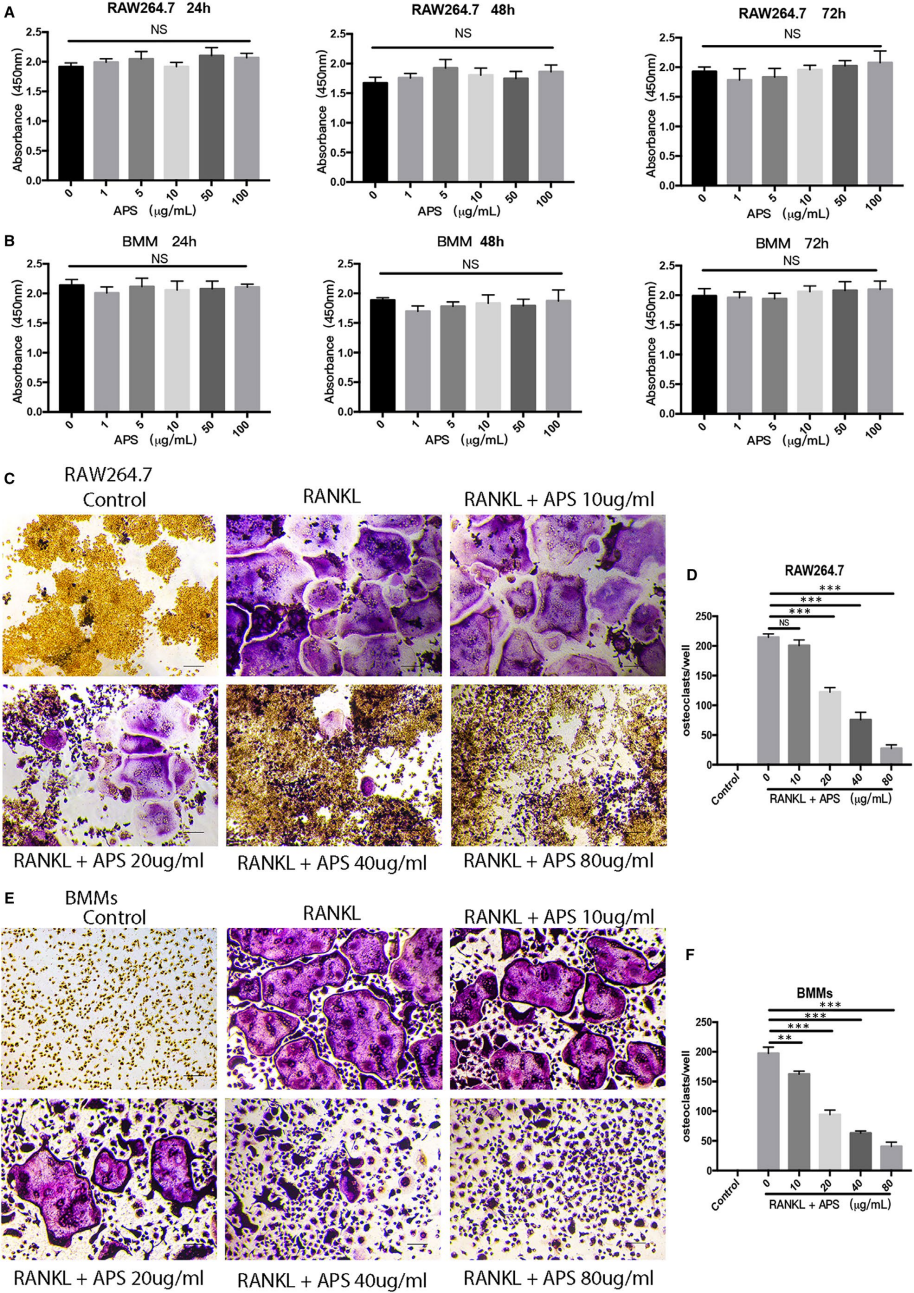
APS suppressed osteoclastogenesis stimulated by RANKL in a concentration‐/time‐dependent fashion in vitro without apparent cytotoxic effects. (A,B) The cell viability of RAW 264.7 cells and BMMs was assessed by using CCK‐8 assay after the treatment of APS (0, 1, 5, 10, 50, 100 µg/mL) for 24, 48 and 72 hours, respectively. (C,E) RAW264.7 and BMMs were cultured in the induction medium containing M‐CSF (25 ng/mL) and RANKL (50 ng/mL) and treated with APS (0, 10, 20, 40, 80 µg/mL) for 5‐6 days. The formation of osteoclast was visualized by TRAP staining. (D,F) The number of TRAP‐positive multinucleated cells (>3 nuclei) in RAW264.7 cells and BMMs. Scale bar, 100 μm. The results are presented as mean ± SD. *P values less than .05, **P values less than .01 and ***P values less than .001 compared with the controls induced by RANKL

The cytotoxic effects of APS on RAW264.7 and BMMs were evaluated by CCK‐8 assays. After treating RAW264.7 and BMMs with APS (0, 1, 5, 10, 50 and 100 mg/mL) for 24 hours, 48 hours and 72 hours, respectively, no anti‐proliferative effects on RAW264.7 (Figure 1A) and BMMs (Figure 1B) were observed after the treatment of APS at 100 mg/mL.

### 3.2 APS inhibited RANKL‐stimulated osteoclast formation and fusion

**FIGURE 2:**
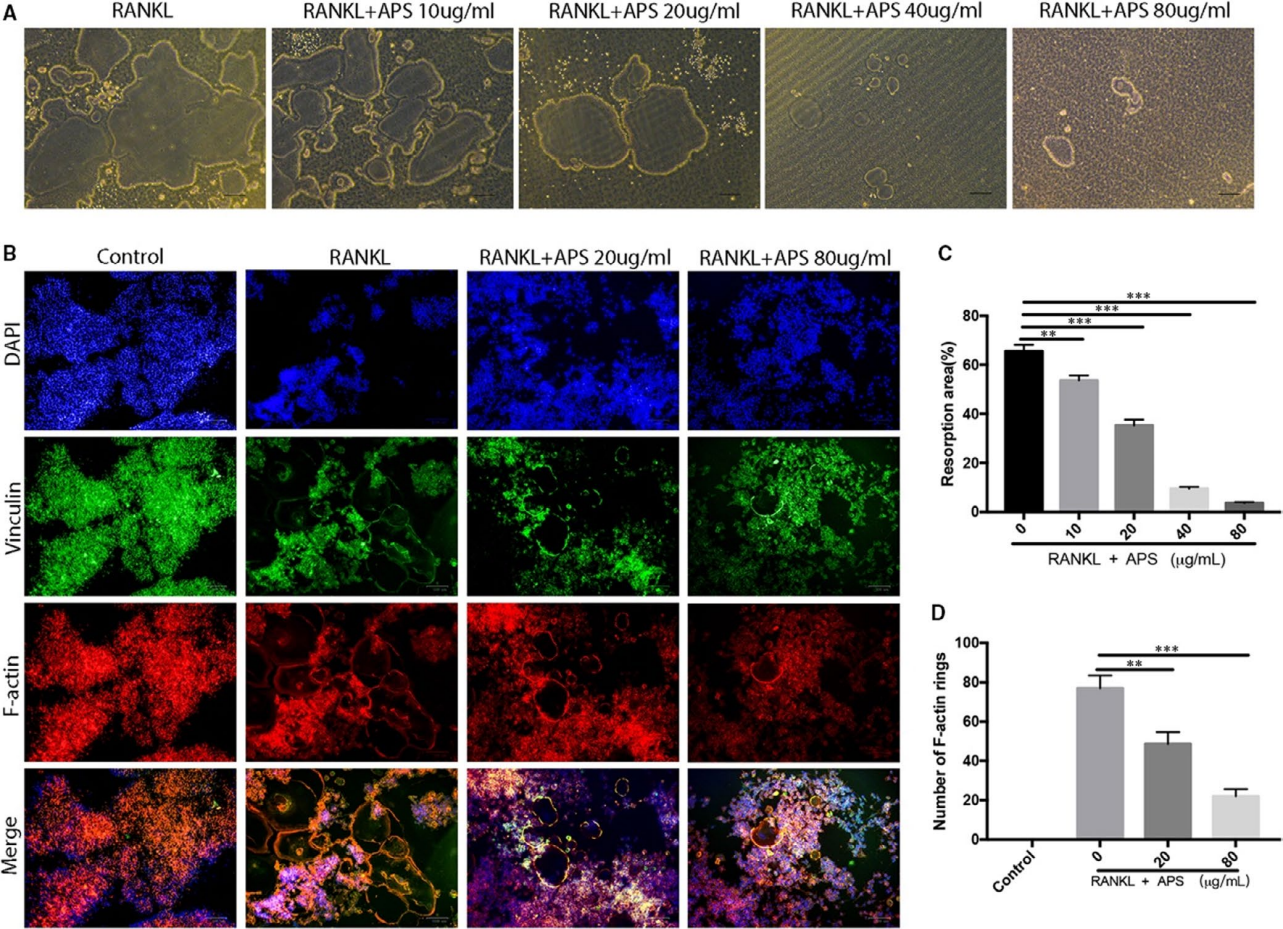
APS attenuated RANKL‐stimulated osteoclastic bone resorption activity in vitro. (A) RAW264.7 cells were placed into osteo assay surface plates and cultured in the induction medium with APS. The images of bone resorption pits were captured by a light microscope after forming mature osteoclasts. (B) RAW264.7 cells were cultured in the induction medium with APS. Representative phalloidin staining images from the actin ring formation were taken by a light microscope after forming a mature osteoclast. (C) The percentage resorption area. (D) The number of intact F‐actin rings. Scale bar, 100 μm. The results are presented as mean ± SD. *P values less than .05, **P values less than .01, and ***P values less than .001 compared with the controls induced by RANKL

First of all, to assess the effects of APS on the osteoclast formation and fusion stimulated by RANKL in vitro, RAW264.7 and BBMs were cultured in complete DMEM and α‐MEM, respectively. Both mediums contained M‐CSF (25 ng/mL) and RANKL (50 ng/mL). The cells were administrated with indicated concentrations of APS (10, 20, 40 and 80 µg/mL) or blank. The TRAP staining results exhibited that APS decreased the number and the area occupied by TRAP‐positive osteoclasts in a concentration‐dependent fashion and apparently reduced the number of nuclei per osteoclast in RAW264.7 (Figure 1C,D) and BMM (Figure 1E,F) cells. Therefore, the low (20 µg/mL) and high (80 µg/mL) doses of APS were chosen for subsequent studies. To explore the effects of APS on the fusion of osteoclasts, the formation of the F‐actin ring was analysed by the performance of actin cytoskeleton/focal adhesion staining. We also noticed the significant inhibition of APS on F‐actin ring formation in the osteoclastogenesis stimulated by RNAKL (Figure 2B,D). Overall, these findings suggest that APS attenuates RANKL‐stimulated formation and fusion of osteoclast in vitro.

### 3.3 APS attenuated bone resorption activity in vitro

Subsequently, a hydroxyapatite resorption experiment was conducted to evaluate the influence of APS on the function of osteoclast. The hydroxyapatite resorption areas shown in Figure 2A,C were apparently reduced after treating osteoclasts with high‐dose APS (80 µg/mL). These data indicated that APS attenuated osteoclastic bone resorption activity in vitro in a concentration‐dependent fashion.

### 3.4 APS reduced RANKL‐stimulated osteoclast‐specific gene levels, osteoclastic differentiation and function‐associated protein levels

**FIGURE 3:**
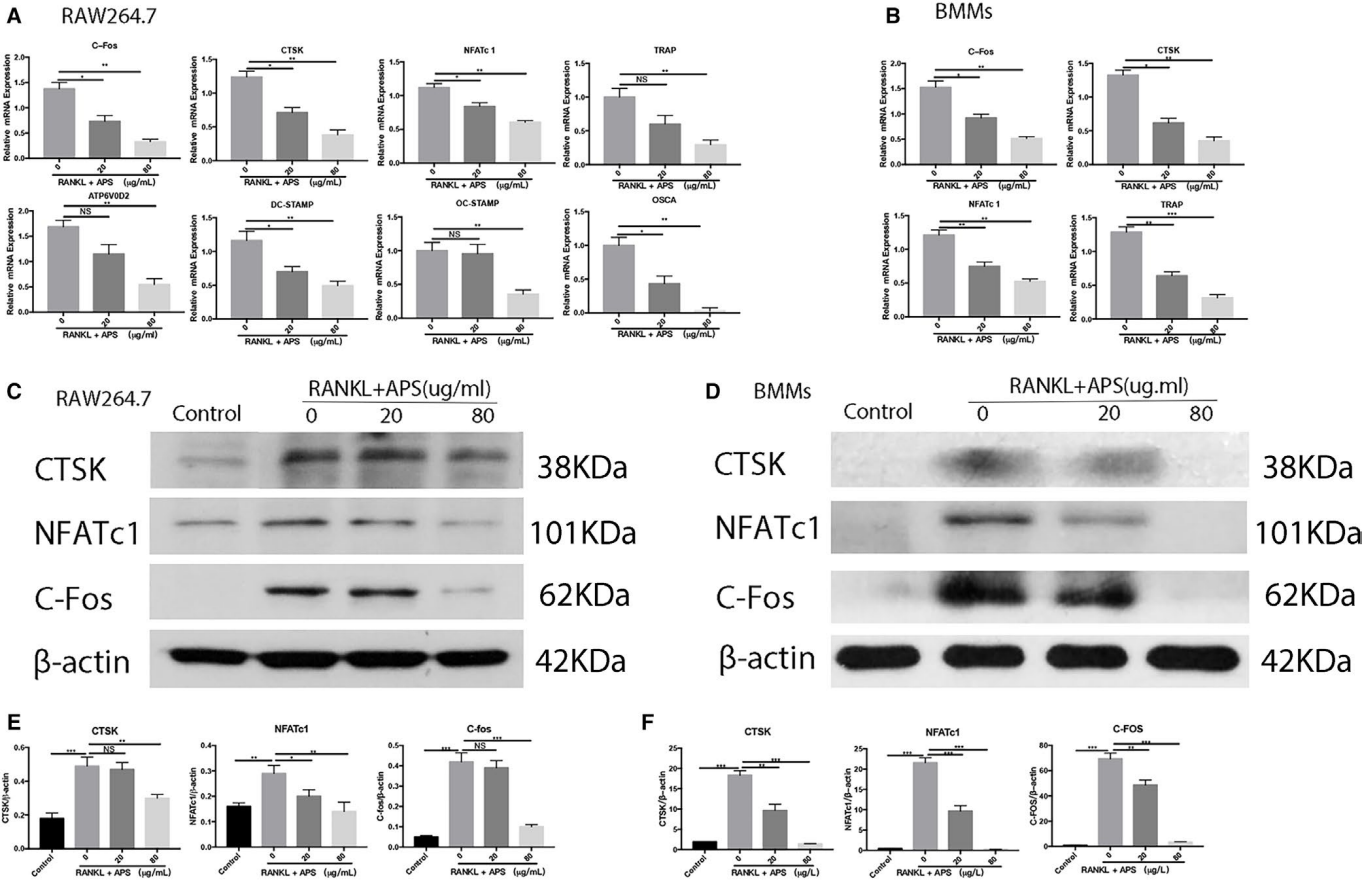
APS down‐regulated the levels of osteoclast‐specific genes and proteins after RANKL induction. Cells were placed into 6‐well plates and cultured with APS for three days (RAW264.7) or five days (BMMs), respectively. (A) The mRNA levels of *NFATc1*, *c‐Fos*, *DC‐STAMP*, *OC‐STAMP*, *CTSK*, *OSCA*, *ATP6V0D2* and *TRAP* in RAW264.7. (B) The mRNA levels of *NFATc1*, *CTSK*, *TRAP* and *c‐Fos* were measured in BMMs. (C,D) The protein expression levels of NFATc1, c‐Fos, CTSK and β‐actin were assessed in RAW264.7 and BMMs. (E and F) The relative levels of NFATc1, c‐Fos and CTSK were analysed by Grey pixel value detection in RAW264.7 and BMMs. Gene expression was normalized to *GAPDH*. β‐actin was served as a reference for protein relative expression. The results are presented as mean ± SD. *P values less than .05, **P values less than .01 and ***P values less than .001 compared with the controls induced by RANKL

We further assessed if APS could inhibit the mRNA expression level of RANKL‐induced osteoclastic marker genes. RAW264.7 and BMMs were respectively stimulated by RANKL and M‐CSF for three days (RAW264.7) or five days (BMMs) followed by the treatment of different concentrations of APS(20 or 80 µg/mL). RT‐qPCR was then conducted to determine the levels of osteoclast marker genes. As demonstrated by RT‐qPCR, the expression levels of RANKL‐induced osteoclastic marker genes, including TRAP, NFATc1, c‐FOS, CTSK, DC‐STAMP, OC‐STAMP, MMP9 and ATP6V0D2, were noticeably down‐regulated in the presence of APS in a concentration‐dependent fashion relative to the control group (Figure 3A,B). Next, to evaluate the effect of APS on RANKL‐induced osteoclastic function‐related protein expression, Western blots were performed to evaluate the most imperative protein of osteoclastic differentiation and function, including NFATc1, c‐FOS and CTSK. APS significantly reduced the protein levels of CTSK, NFATc1 and c‐Fos in RAW264.7 (Figure 3C,E) and BMMs (Figure 3D,F) stimulated by RANKL and M‐CSF.

### 3.5 APS reduced the LPS‐related differentiation and function of osteoclast in vitro

**FIGURE 4:**
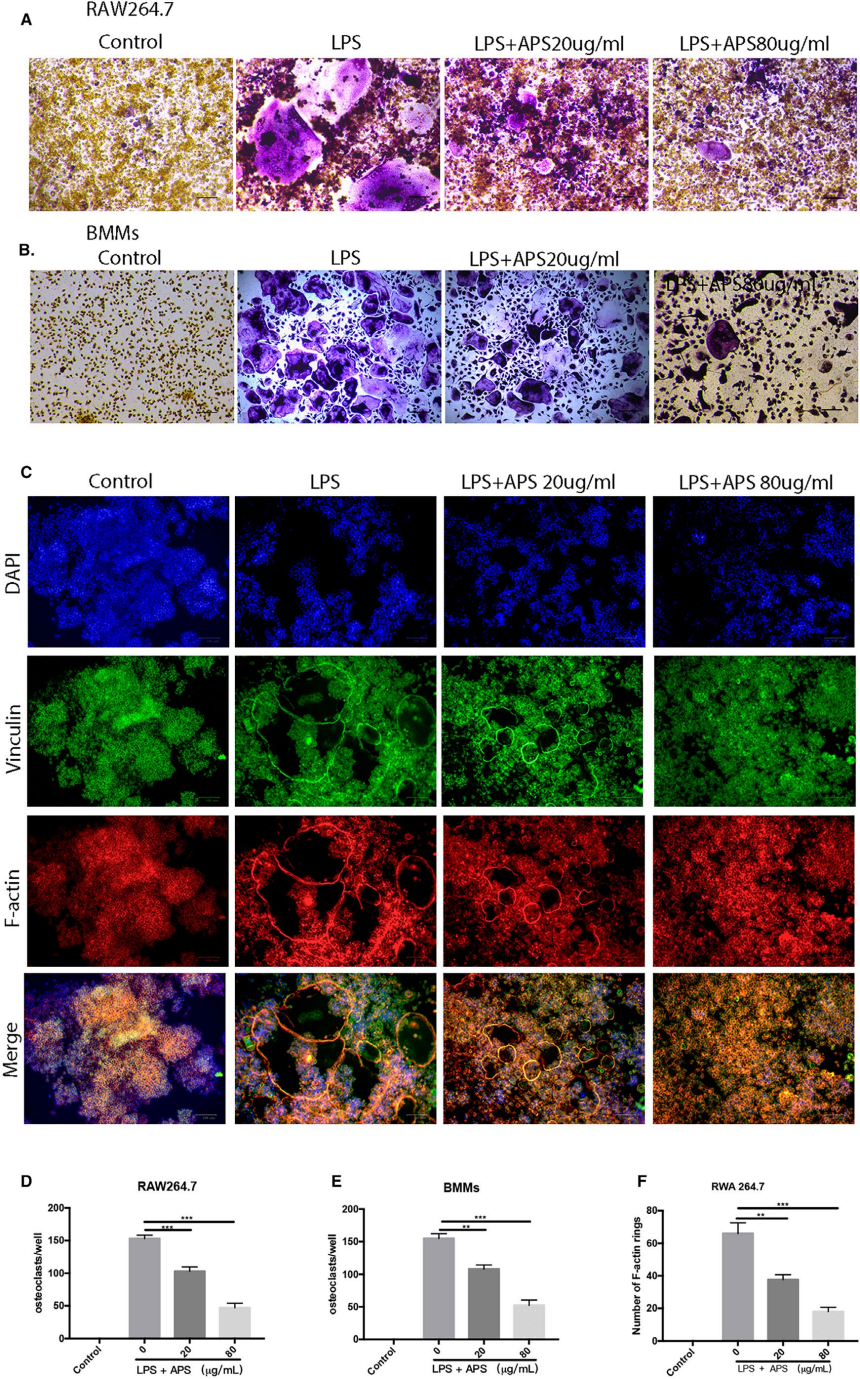
APS inhibited LPS‐stimulated osteoclastogenesis in a concentration‐/time‐dependent fashion in vitro without apparent cytotoxic effects. (A,B) RAW264.7 and BMMs were cultured in an induction medium (100 ng/mL LPS) and administrated with APS (0, 10, 20, 40, 80 μg/mL) for 5‐6 days. Osteoclast formation was visualized by TRAP staining. (C) RAW264.7 cells were cultured in an induction medium (100 ng/mL LPS) and treated with APS. Representative phalloidin staining images from the actin ring formation were taken by microscope after the formation of mature osteoclast. (D,E) The number of TRAP‐positive multinucleated cells (>3 nuclei) in RAW264.7 and BMMs. (F) The number of intact F‐actin rings. Scale bar, 100 μm. The results are presented as mean ± SD. *P values less than .05, **P values less than .01 and ***P values less than .001 compared with the controls induced by LPS

**FIGURE 5:**
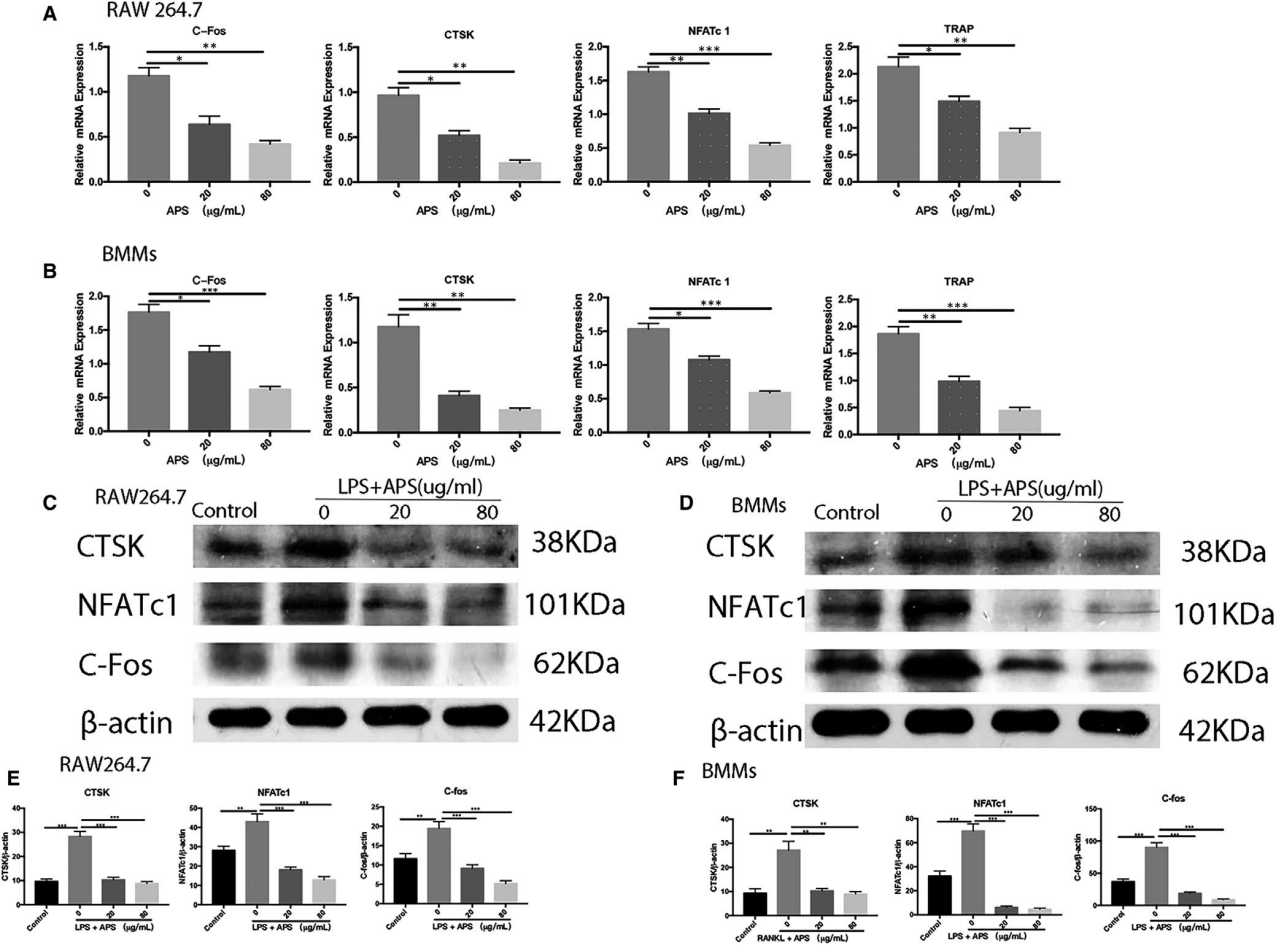
APS down‐regulated the levels of osteoclast‐specific genes and proteins associated with LPS induction. The cells were placed into 6‐well plates and cultured with APS for three days (RAW264.7) or five days (BMMs), respectively. (A,B) The mRNA levels of *NFATc1*, *c‐Fos*, *CTSK* and *TRAP* in RAW264.7 and BMMs (normalized to *GAPDH*). (C,D). The protein levels of NFATc1, c‐Fos, CTSK and β‐actin were measured in RAW264.7 and BMMs. (E,F) The levels of NFATc1, c‐Fos and CTSK in RAW264.7 and BMMs were analysed by Grey pixel value detection. β‐actin was applied as a reference. The results are presented as mean ± SD. *P values less than .05, **P values less than .01 and ***P values less than .001 compared with the controls induced by RANKL

To evaluate if APS could influence LPS‐associated osteoclastogenesis, RAW264.7 and BMMs were cultured into 96‐well plates and stimulated by M‐CSF and RANKL for 24 hours. After that, the cells were administrated with LPS (100 ng/mL) and APS (10, 20, 40 and 80 µg/mL). The findings indicated that APS decreased the LPS‐stimulated formation and differentiation of osteoclast in RAW264.7 (Figure 4A,D) and BMMs (Figure 4B,E) in a dose‐dependent fashion, which is consistent with the RANKL‐induced studies abovementioned. We also observed that APS significantly reduced F‐actin ring formation during LPS‐induced osteoclastogenesis (Figure 4C,F). Moreover, we evaluated the inhibitory effects of APS on levels of osteoclast‐related genes induced by LPS and found that APS dose‐dependently prevented LPS‐induced overexpression of osteoclastic specific marker genes (NFATc1, CTSK, TRAP and c‐FOS), which is consistent with the non‐inflammatory environment (Figure 5A,B). Besides, Western blotting results also demonstrated that APS effectively reduced the levels of specific proteins associated with osteoclast, including CTSK, NFATc1 and c‐Fos in RAW264.7 (Figure 5C,E) and BMMs (Figure 5D,F). Overall, our findings suggest that APS dose‐dependently inhibits the differentiation and formation of LPS‐induced osteoclasts.

### 3.6 APS suppressed osteoclastogenesis through the MAPK signalling pathways

**FIGURE 6:**
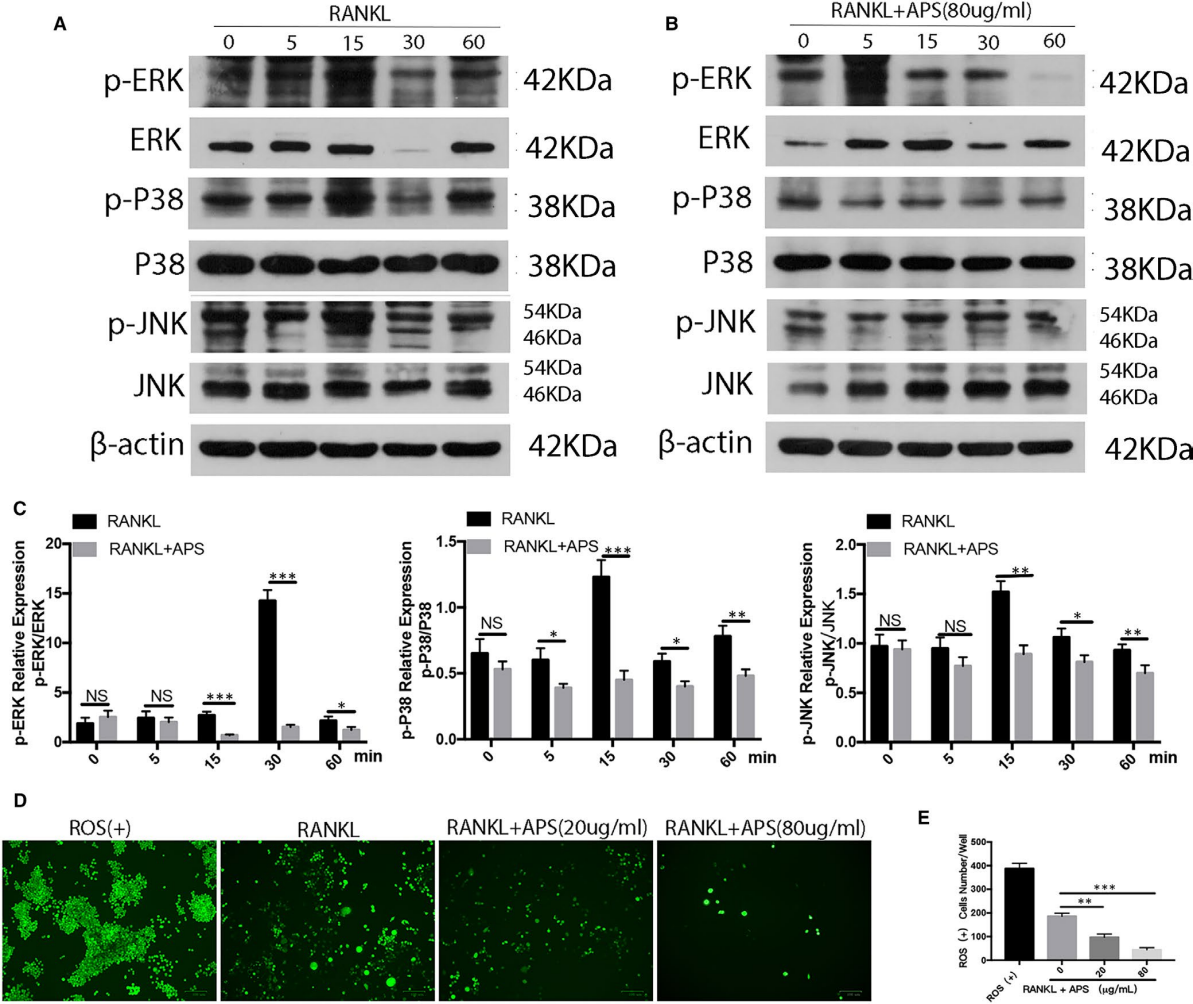
APS inhibited osteoclastogenesis via the MAPK signalling pathways. (A) RAW264.7 cells were stimulated in the complete induction medium in the absence of APS for 0, 5, 15, 30 and 60 minutes, respectively. Western blots were performed with specific antibodies against β‐actin, p‐JNK, JNK, p‐ERK, ERK, p‐p38 and p38. (B) BMMs cultured in the complete induction medium were administrated with APS for 0, 5, 15, 30 and 60 minutes, respectively. Western blots were performed with specific antibodies against β‐actin, p‐JNK, JNK, p‐ERK, ERK, p‐p38 and p38. (C) The ratios of p‐ERK/ERK, p‐P38/P38 and p‐JNK/JNK were analysed by Grey pixel value detection. (D) The ROS levels were determined in RAW264.7. (E) The levels of ROS were measured. Scale bar, 100 μm. The results are presented as mean ± SD. *P values less than .05, **P values less than .01 and ***P values less than .001 compared with the controls induced by LPS

The three primary subfamilies of MAPKs (p38, ERK1/2 and JNK), downstream of RANK signalling, are well known to play essential roles in osteoclast development. To better understand the underlying mechanism of the inhibitory effects of APS on the differentiation of osteoclast, we examined the impact of APS on RANKL‐stimulated pathways by Western blot. RAW264.7 induced by RANKL and M‐CSF were treated with APS (80 µg/mL) or blank (PBS) for 0, 5, 15, 30 and 60 minutes to assess the MAPK signalling pathway activation. At the same time, the phosphorylation levels of p38, ERK and JNK were maximized within 15, 30 and 30 minutes in the control group after RANKL stimulation, respectively (Figure 6A). It was noted that the treatment with APS significantly inhibited the phosphorylation trend of these signalling pathways (Figure 6A‐C). Therefore, the results showed that APS reduced the differentiation and function of RANKL‐stimulated osteoclasts by attenuating the activation of three MAPK (ERK, JNK and p38) signalling pathways.

### 3.7 APS inhibited intracellular ROS production stimulated by RANKL

As mentioned earlier, APS is an antioxidant. Therefore, to explore the underlying mechanism of APS‐dependent osteoclastogenesis inhibition, the impact of APS on RANKL‐induced intracellular ROS production was evaluated. APS decreased the intensity of ROS staining and the ROS‐positive cell numbers in a dose‐dependent fashion (Figure 6D,E). Therefore, we consider that APS may inhibit osteoclast formation by inhibiting ROS production.

### 3.8 APS protected LPS‐stimulated osteolysis in a mouse model of the calvaria

**FIGURE 7:**
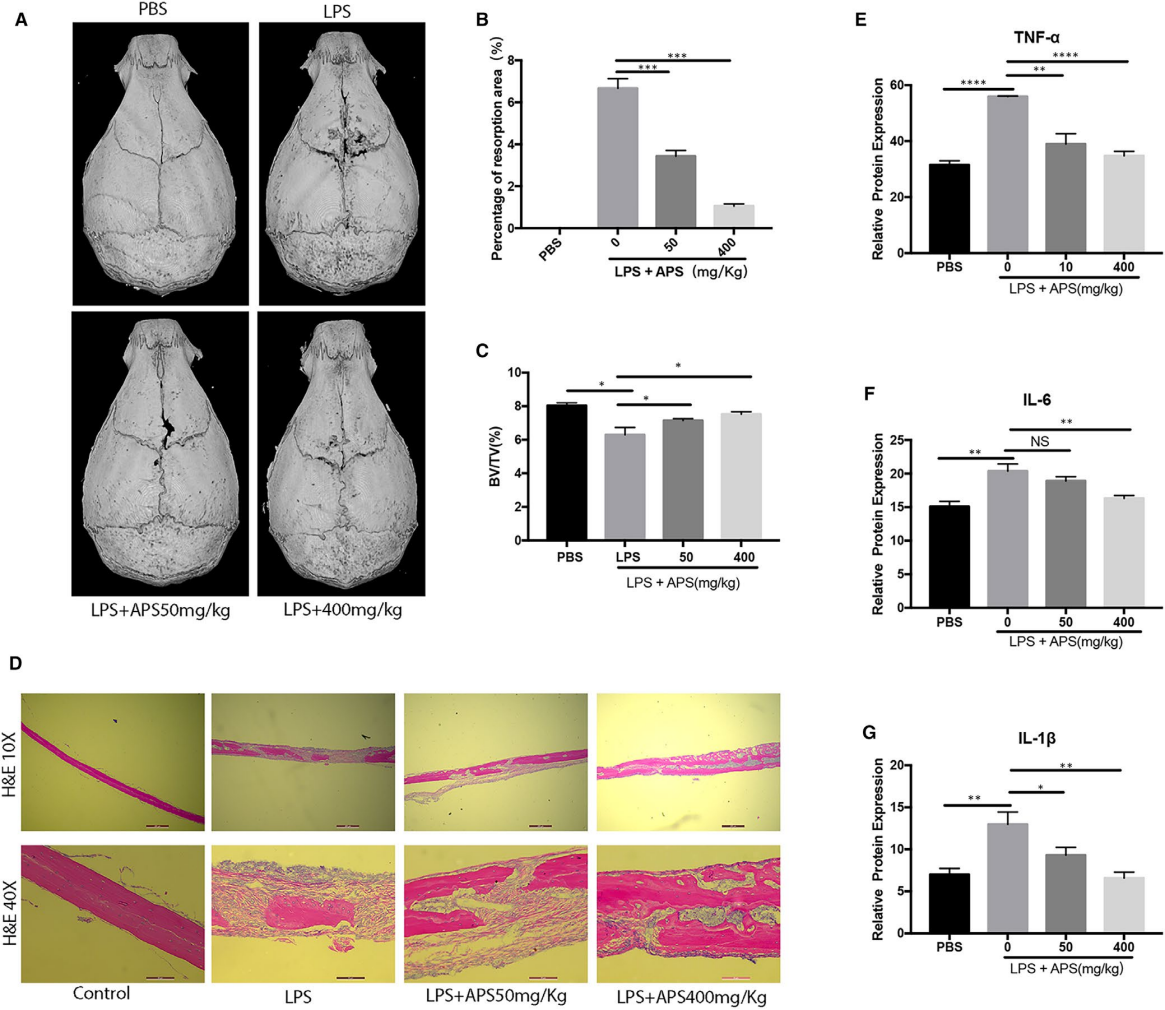
APS suppressed osteolysis in vivo. (A) Micro‐CT scanning and 3D reconstruction of the outer surface of calvaria in the blank control, LPS control, low‐dose APS‐treated and high‐dose APS‐treated groups. Quantitative analyses were performed to determine the percentage of the resorption area of the whole calvariae (B) and the BV/TV% of the whole calvariae (C). (D) H&E staining was performed on the mouse calvariae collected from the blank control, LPS control, low‐dose APS‐treated and high‐dose APS‐treated groups. (E‐G) The levels of TNF‐α, IL‐1β and IL‐6 in mouse serum were assessed by ELISA. Scale bar, 100 μm. The results are presented as mean ± SD. *P values less than .05, **P values less than .01 and ***P values less than .001 compared with the controls induced by LPS

To investigate if APS can reduce LPS‐related bone destruction in vivo, we further established a mouse model of LPS‐induced inflammatory osteolysis. LPS (5 mg/kg) was injected along with APS (low: 50 mg/kg; high: 400 mg/kg) or blank control (PBS) into a sagittal suture in the mouse skull. Micro‐CT scanning and 3D image reconstruction were applied to assess the levels of bone loss. As shown in 3D image reconstruction, an extensive trabecular bone loss was shown in the LPS control group compared with the blank control group (Figure 7A,B). In contrast, low‐dose and high‐dose APS treatments effectively ameliorated the occurrence of osteolytic bone loss (Figure 7A,B). On the other hand, quantitative analysis of bone parameters confirmed that there were significant reductions in BV/TV (Figure 7C) in the LPS control group, while APS treatments inhibited the decrease in LPS‐induced BV/TV (Figure 7C). The histological analysis results (H&E staining) confirmed that APS prevented LPS‐induced osteolysis (Figure 7D), consistent with the micro‐CT scan results. In addition, an ELISA kit was applied to analyse the expression levels of IL‐6, TNFα and IL‐1β. The results exhibited that APS observably reduced the abovementioned pro‐inflammatory cytokines' production (Figure 7E‐G) in mouse serum. In conclusion, these data support that APS prevents bone loss from LPS stimulation in vivo.

## 4 DISCUSSION

Chronic gram‐negative bacterial bone diseases, including infection of orthopaedic implants, periodontitis, septic arthritis, septic arthritis and osteomyelitis, are often caused by enhanced osteoclastic activity and osteolysis.^22^, ^23^ At present, it is evident that the molecular mechanism of bacteria‐induced osteolysis is related to the activation of immune cells by bacterial endotoxin, which ultimately leads to the formation of osteoclasts.^12^, ^24^, ^25^ Unfortunately, to date, available treatment approaches for inflammatory osteolysis are still limited. It is well known that bisphosphonate, an antiresorptive agent with a definite curative effect, is widely applied to treat osteolytic bone diseases,^26^, ^27^ while it may result in some additional complications, such as osteonecrosis of the jaw and atypical fractures, eventually causing orthopedists' confusion.^26^, ^27^ Hence, the exploration of novel and effective anti‐osteolysis agents is still highly required.

LPS, a classic potent stimulus of the immune system, is a significant endotoxin of gram‐negative bacteria.^7^, ^8^ Moreover, LPS has been known as a potent stimulus of the progression of inflammatory osteolysis for a long time, so that it is widely used to establish an animal inflammatory bone erosion model.^28^, ^29^ In this study, LPS was utilized to cause inflammatory osteolysis in a mouse model of calvaria. As the main extractive of Astragali Radix, APS has been widely used for treating inflammatory diseases.^21^, ^30^, ^31^ As osteoclast plays a critical role in inflammatory osteolysis,^1^, ^32^ the current study aimed at investigating if APS has the ability to inhibit osteoclastogenesis in murine models of inflammatory osteolysis. We found that APS is a practical suppressor to prevent osteoclast formation in vitro by reducing the number and area of TRAP‐positive multinucleated cells. Moreover, we further demonstrated that APS administration decreased the mRNA levels of various osteoclast marker genes, such as *NFATc1*, *TRAP*, *ATP6V0D2*, *CTSK*, *c‐Fos*, *DC‐STAMP*, *OC‐STAMP* and *OSCA*. The bone resorption assay in vitro showed consistent results regarding the inhibitory effect of APS on the formation of osteoclast. Additionally, APS treatment significantly decreased the production of IL‐6, IL‐1β and TNF‐α in vitro relative to the LPS group and effectively prevented osteolytic bone loss associated with LPS stimulation in vivo.

**FIGURE 8:**
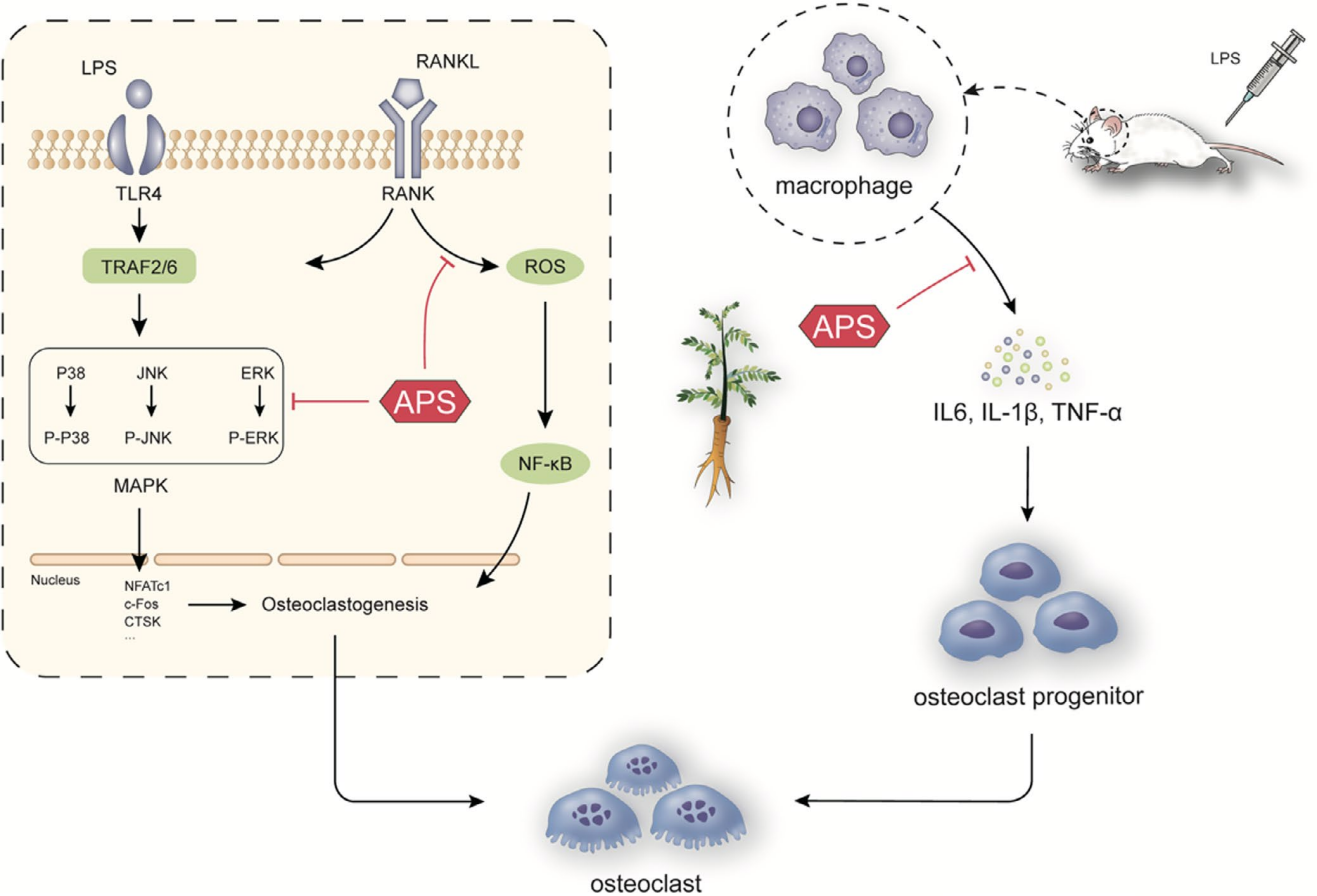
Schematic diagram of the suppressing influence of APS on the formation of osteoclast and activity of bone resorption

The underlying mechanism of APS's inhibition on osteoclast formation was further investigated. RANKL is an indispensable critical cytokine that participates in osteoclast formation, differentiation and function.^29^, ^33^ After stimulation by RANKL, TRAF2/6 signalling is activated by RANK, resulting in the phosphorylation of three MAPK pathways (JNK, P38 and ERK) mediated by MEK1/2, MKK7 and MKK6.^28^, ^33^, ^34^ Furthermore, an increased level of c‐Fos is essential for the production of NFATc1 to regulate osteoclast differentiation.^35^, ^36^ NFATc1 is recognized as a well‐characterized essential transcription factor required to activate osteoclastic‐specific genes, including *CTSK*, *TRAP*, *DC‐STAMP*, *MMP9* and *ATP6V0D2*. These genes play substantial roles in the proliferation, differentiation, maturation and osteoclastic bone resorption of osteoclasts.^35^, ^37^, ^38^ Our study shows that APS suppresses osteoclast formation and osteolysis via its broad‐spectrum inhibition on MAPK signalling pathways (ERK, JNK and p38) stimulated by RANKL in line with the results of APS suppressed osteolysis observed in vivo. Together with the inhibitory effects of APS on MAPK pathways, the levels of genes and proteins associated with osteolysis were reduced remarkably. The activation of downstream marker genes was also restrained in a concentration‐dependent fashion. On the other hand, we also confirmed that APS inhibited intracellular ROS production stimulated by RANKL. ROS are essential for cell signalling and other physiological functions, including cell proliferation regulation, metabolism, apoptosis, survival, differentiation and migration.^39^, ^40^, ^41^ Several recent studies have shown that the osteoclast differentiation associated with RANKL induction is accompanied by increasing the intracellular ROS production while reducing the intracellular ROS levels can significantly inhibit osteoclast differentiation function.^40^, ^41^, ^42^, ^43^ Therefore, ROS are considered an essential component that regulates osteoclastic differentiation and function. The ROS‐induced osteoclast differentiation mechanism may be related to the effects of ROS on activating the downstream NF‐kB signalling pathway and further activate osteoclast‐related genes, such as *NFATc1*.^41^, ^44^, ^45^ In summary, our findings demonstrate that APS acts as an inhibitor to reduce the activation of MAPK and ROS signalling pathways during osteoclastogenesis associated with RANKL induction (Figure 8).

Toll‐like receptors (TLRs) family, a panel of conserved pattern‐recognition receptors (PRR), are the type I transmembrane proteins receptor activated by various pathogen‐related molecular patterns, thus activating multiple signals.^46^, ^47^ TLR4 belongs to the TLR family that is recognized and up‐regulated by LPS.^48^, ^49^ Along with the activation of TLR4, an increased expression of TRAF6 results in the activation of the MAPK pathway and ultimately leads to increased levels of marker genes, such as *c‐Fos*, *CTSK* and *NFATc1*, associated with osteoclast differentiation.^13^, ^50^ Therefore, in view of the mechanism, APS inhibits osteoclastogenesis and bone resorption by reducing the MAPK signalling pathway and may also inhibit LPS‐induced osteoclast formation by inhibiting MAPK signalling. In addition, LPS was found to stimulate the expression of multiple factors related to inflammation, including TNF‐α, IL‐6 and IL‐1β, thereby promoting osteoclastogenesis by activating NF‐kB and MAPKs signal pathways.^9^, ^11^, ^12^ The in vivo studies demonstrate that APS can inhibit the serum levels of LPS‐induced pro‐inflammatory factors. Overall, these results suggest that APS inhibits the activation of MAPK signalling pathways and the levels of pro‐inflammatory factors during LPS‐related osteoclastogenesis.

## 5 CONCLUSIONS

In conclusion, the current study proves that APS inhibits osteoclastogenesis and osteoclast function induced by RANKL/LPS in vitro and LPS‐related inflammatory osteolysis in vivo. It has been clarified that APS exhibits suppressive effects on the MAPK and ROS signalling pathways, ROS production and the downstream factors responsible for osteoclastogenesis. In addition, APS also reduces LPS‐induced inflammatory osteolysis in vivo by decreasing inflammatory factors' production. Therefore, our findings indicate that APS has the potential to become a therapeutic candidate for osteolytic‐related diseases.

## CONFLICT OF INTEREST

The authors declare that they have no conflict of interest.

## AUTHOR CONTRIBUTION

**Jianye Yang:** Conceptualization (equal); Data curation (lead); Formal analysis (equal); Investigation (equal); Methodology (lead); Project administration (equal); Software (equal); Supervision (equal); Writing‐original draft (lead); Writing‐review & editing (lead). **Leilei Qin:** Data curation (equal); Formal analysis (equal); Investigation (equal); Methodology (equal); Validation (equal); Visualization (equal); Writing‐review & editing (equal). **Jiaxing Huang:** Data curation (equal); Formal analysis (equal); Investigation (equal); Methodology (equal); Project administration (equal); Resources (equal). **Yuwan Li:** Conceptualization (equal); Data curation (equal); Formal analysis (equal); Investigation (equal); Methodology (equal); Project administration (equal); Writing‐review & editing (equal). **Sha Xu:** Conceptualization (equal); Data curation (equal); Formal analysis (equal); Investigation (equal); Methodology (equal); Project administration (equal); Supervision (equal). **Hai Wang:** Data curation (equal); Formal analysis (equal); Methodology (equal); Project administration (equal); Supervision (equal); Validation (equal). **Sizheng Zhu:** Conceptualization (equal); Formal analysis (equal); Investigation (equal); Project administration (equal); Resources (equal). **Jiawei Wang:** Conceptualization (equal); Data curation (equal); Formal analysis (equal); Project administration (equal); Resources (equal); Software (equal); Supervision (equal). **Bo Zhu:** Conceptualization (equal); Data curation (equal); Formal analysis (equal); Project administration (equal); Resources (equal); Software (equal); Visualization (equal). **Feilong Li:** Conceptualization (equal); Data curation (equal); Methodology (equal); Software (equal); Supervision (equal). **Wei Huang:** Funding acquisition (equal); Project administration (equal); Resources (equal); Software (equal); Supervision (equal); Validation (equal); Visualization (equal). **Xuan Gong:** Funding acquisition (equal); Project administration (equal); Resources (equal); Software (equal); Supervision (equal); Validation (equal); Visualization (equal). **Ning Hu:** Conceptualization (equal); Data curation (equal); Formal analysis (equal); Funding acquisition (equal); Investigation (equal); Project administration (equal); Supervision (equal).

## Data Availability

The data that support the findings of this study are available from the corresponding author upon reasonable request.
