# Correction to ‘Astragalus polysaccharide attenuates LPS‐related inflammatory osteolysis by suppressing osteoclastogenesis by reducing the MAPK signalling pathway’

**DOI:** 10.1111/jcmm.71429

**Published:** 2026-11-05

J. Yang , L. Qin , J. Huang , et al., “Astragalus polysaccharide attenuates LPS‐related inflammatory osteolysis by suppressing osteoclastogenesis by reducing the MAPK signalling pathway,” Journal of Cellular and Molecular Medicine 25, no. 14 (2021): 6800–6814. 10.1111/jcmm.16683 PMC827812434080298.

In the article, the authors identified an error in Figure 2A. The correct image is shown below. The authors confirm all results and conclusions of this article remain unchanged.

**FIGURE 2:**
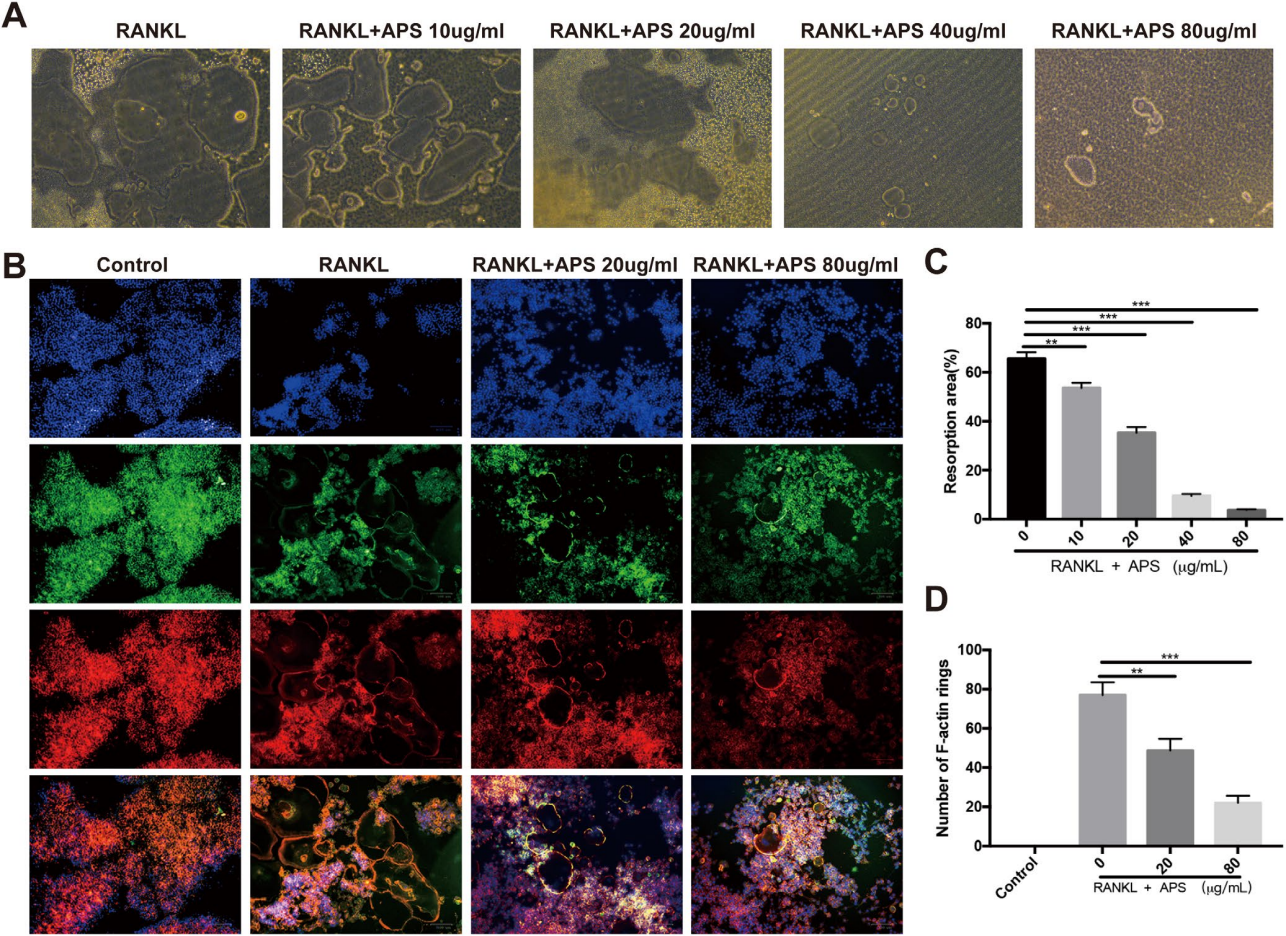
APS attenuated RANKL‐stimulated osteoclastic bone resorption activity in vitro. (A) RAW264.7 cells were placed into osteoassay surface plates and cultured in the induction medium with APS. The images of bone resorption pits were captured by a light microscope after forming mature osteoclasts. (B) RAW264.7 cells were cultured in the induction medium with APS. Representative phalloidin staining images from the actin ring formation were taken by a light microscope after forming a mature osteoclast. (C) The percentage resorption area. (D) The number of intact F‐actin rings. Scale bar, 100 μm. The results are presented as mean ± SD. *p values <0.05, **p values <0.001, and ***p values <0.001 compared with the controls induced by RANKL.

We apologize for the error.

